# Epidemiology and survival outcomes of patients with orbital region non-cutaneous squamous cell carcinoma: a population-based analysis

**DOI:** 10.3389/fonc.2023.1152337

**Published:** 2023-05-05

**Authors:** Lin-feng He, Pei Mou, Rui-li Wei

**Affiliations:** Department of Ophthalmology, Changzheng Hospital of Naval Medicine University, Shanghai, China

**Keywords:** orbital region, non-cutaneous squamous cell carcinoma, SEER, epidemiology, prognosis, survival

## Abstract

**Background:**

Non-cutaneous squamous cell carcinoma (ncSCC) of the orbital region is very rare. Thus, its epidemiological characteristics and prognosis are poorly understood. The aim of the study was to assess the epidemiological characteristics and survival outcomes of ncSCC of the orbital region.

**Methods:**

Incidence and demographic data on ncSCC of the orbital region were extracted from the Surveillance, Epidemiology, and End Results (SEER) database and analyzed. The chi-square test was used to calculate the differences between groups. Univariate and multivariate Cox regression analyses were performed to determine the independent prognostic factors for disease-specific survival (DSS) and overall survival (OS).

**Results:**

The overall incidence of ncSCC in the orbital region from 1975 to 2019 was 0.68/1,000,000, and the incidence showed an increasing trend during this period. A total of 1,265 patients with ncSCC of the orbital region (mean age, 65.3 years) were identified in the SEER database. Of these, 65.1% were aged ≥60 years, 87.4% were White, and 73.5% were male. The conjunctiva (74.5%) was the most common primary site, followed by the orbit (12.1%), lacrimal apparatus (10.8%), and overlapping lesion of the eye and adnexa (2.7%). Multivariate Cox regression analysis revealed that age, primary site, SEER summary stage, and surgery were independent prognostic factors for DSS, whereas age, sex, marital status, primary site, SEER summary stage, and surgery were independent prognostic factors for OS.

**Conclusions:**

The incidence of ncSCC in the orbital region has increased over the past 40 years. It usually affects White people, men, and people aged ≥60 years, and its most common site is the conjunctiva. Orbital SCC has worse survival outcomes than SCC of other sites in the orbital region. Surgery is the independent protective treatment for ncSCC of the orbital region.

## 1 Introduction

**Figure 1:**
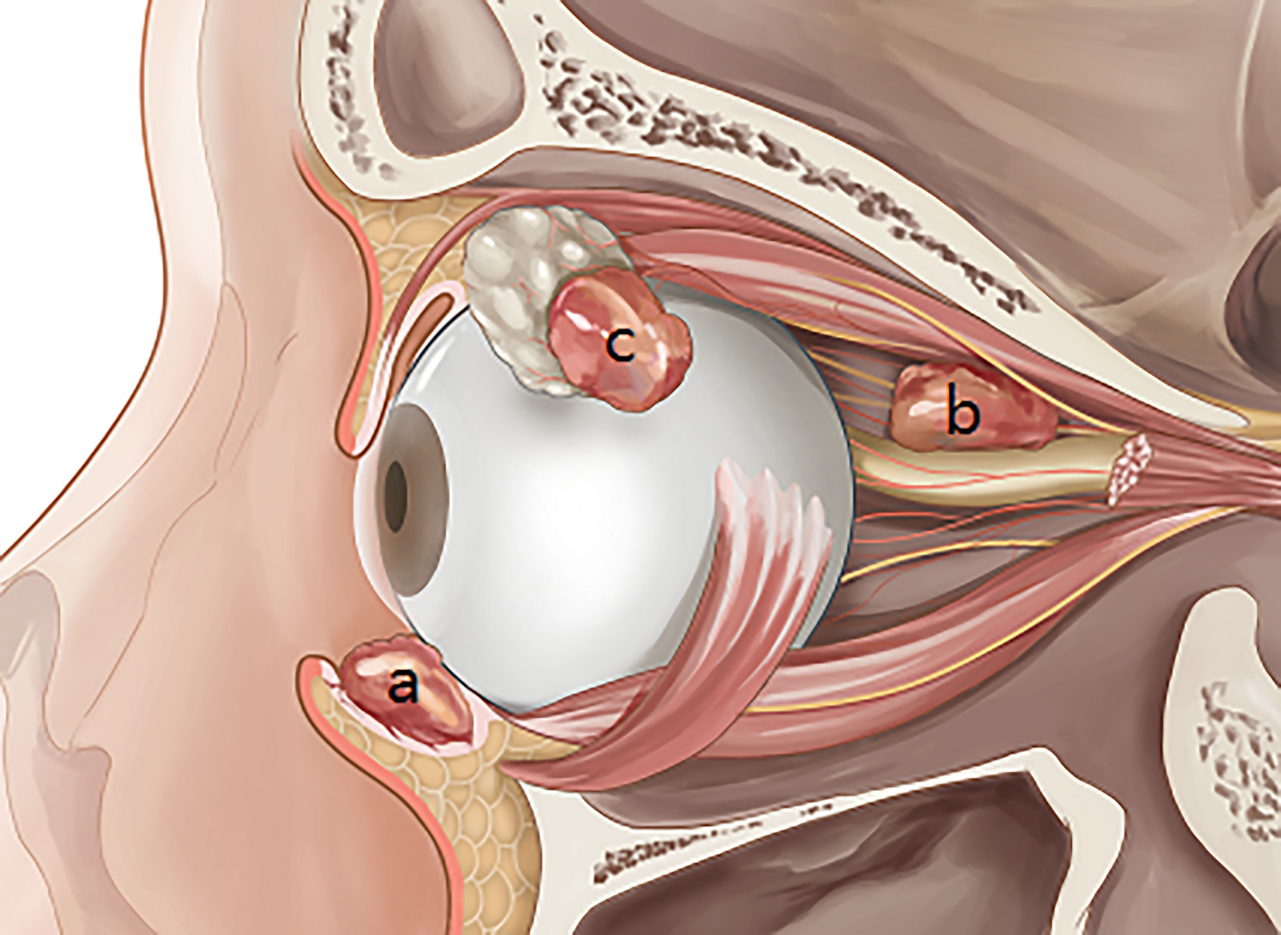
Squamous cell carcinoma in the main non-cutaneous sites of orbital region including conjunctiva **(A)**, orbit **(B)**, and lacrimal apparatus **(C)**.

**Figure 2:**
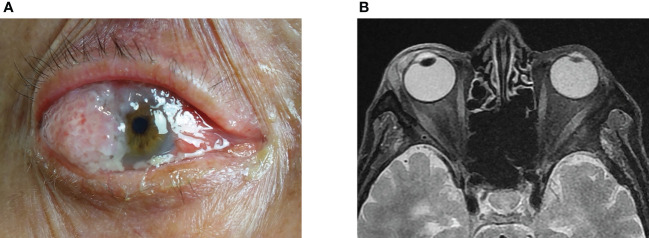
Clinical features of conjunctiva SCC showed a red, raised papilliform lesion on the conjunctiva that extended onto the cornea, with accompanying sticky mucoid discharge **(A)**. The orbital magnetic resonance imaging demonstrated a flat tumor in front of the eyeball **(B)**. SCC, squamous cell carcinoma.

Squamous cell carcinoma (SCC) is the second most common malignancy of the eyelid and has a favorable prognosis with timely treatment (1, 2). SCC can develop in other non-cutaneous sites around the eye (**Figure 1**), including the conjunctiva (**Figure 1A**), orbit (**Figure 1B**), and lacrimal apparatus (**Figure 1C**). However, SCC of these ocular structures is rare compared with SCC of the eyelid (3–6). Conjunctival SCC (**Figures 2A, B**) penetrates the basement membrane, develops in the stroma of the conjunctiva, and potentially extends into the eye, orbit, and sinuses (7). Conjunctival SCC often presents as a slow-growing solitary or diffuse tumor, and its most common clinical symptoms are redness, foreign body sensation, and irritation (3). Orbital SCC is mainly derived from the paranasal sinuses, periocular skin, and nasopharynx or metastases from other sites. Primary orbital SCC is extremely rare, with only a few cases reported to date (8–10). Regarding the lacrimal apparatus, the most common lacrimal gland epithelial malignancy is adenoid cystic carcinoma, followed by pleomorphic adenocarcinoma. However, SCC is the most commonly reported rare tumor of the lacrimal gland (11, 12) and the most common tumor of the lacrimal drainage system. SCC of the lacrimal drainage system usually presents as epiphora, and its symptoms resemble those of inflammatory disease (5, 6, 13). SCC of the lacrimal gland and lacrimal drainage system is rare and potentially life-threatening (5, 13).

The incidence of conjunctival SCC has increased over the years owing to an increase in human papillomavirus infections and ultraviolet light exposure (14). However, the available information on the epidemiological characteristics of non-cutaneous SCC (ncSCC) of the orbital region is limited. Thus, the aim of this study was to describe the epidemiology, characteristics, and prognosis of ncSCC of the orbital region using data from the Surveillance, Epidemiology, and End Results (SEER) database. The SEER database covers approximately 28% of the population in the United States; includes data on the incidence, demographic characteristics, pathology, and prognoses of tumors; and is particularly important for the analysis of rare cancers (15).

## 2 Methods

### 2.1 Data source and study population

This retrospective cohort study was conducted using epidemiological and clinicopathological information extracted from the SEER database. The SEER*Stat software (version 8.4.0.1) was used for the extraction of data from the database. To increase the representativeness of our study, we extracted the data of eligible patients diagnosed between 1975 and 2018 from the SEER 18, 13, and 9 Registries (16–18). The inclusion criteria were as follows: 1) SCC diagnosis (International Classification of Diseases for Oncology histological codes 8050-8089); 2) diagnoses of SCC of a non-cutaneous structure in the orbital region indicated using the site-specific codes C69.0 (conjunctiva), C69.5 (orbit), C69.6 (lacrimal apparatus), and C69.8 (overlapping lesion of the eye and adnexa); 3) diagnosis confirmed through microscopic confirmation; and 4) available data on active follow-up. The exclusion criteria were as follows: 1) diagnosis through an autopsy or death certificate, 2) unknown survival or survival months = 0, 3) unknown SEER summary stage, and 4) prior malignancy diagnosis.

We extracted data on the following variables from the SEER database: age, sex (male and female), race (White and others), year of diagnosis, primary site, marital status (married and others), histological subtype, surgery (performed, no/unknown), radiotherapy (performed, no/unknown), chemotherapy (performed, no/unknown), and SEER summary stage (localized, regional, or distant). The primary endpoints of this study were disease-specific survival (DSS) and overall survival (OS). The annual incidence rates of ncSCC of the orbital region from 1975 to 2019 were extracted from SEER 9 Registries, and all the rates were adjusted to the 2000 United States standard population (19). Given that the SEER database is publicly available and we had access to it after completing an agreement form, approval from an institutional review board was not required for this retrospective cohort study.

### 2.2 Statistical analyses

The age-adjusted incidence rates of ncSCC of the orbital region were calculated per 1,000,000 persons using the SEER*Stat software (version 8.4.0.1). The 95% confidence interval (CI) and annual percentage change (APC) of the incidence rates were calculated as well. Statistical comparisons of age, sex, race, and primary site were performed using the chi-squared test. All the clinicopathological variables were converted into categorical variables and are expressed as frequencies. DSS and OS were analyzed using the Kaplan–Meier method, and differences were calculated using the log-rank test. Univariate and multivariate Cox proportional hazards models were used for survival analysis. Statistical significance was set at p < 0.05. Statistical analyses were conducted using SPSS (version 18.0; SPSS version 26.0; IBM SPSS Statistics, IBM Corporation, Armonk, NY, USA) and R software (version 4.2.2).

## 3 Results

### 3.1 Incidence of ncSCC of the orbital region

**Figure 3:**
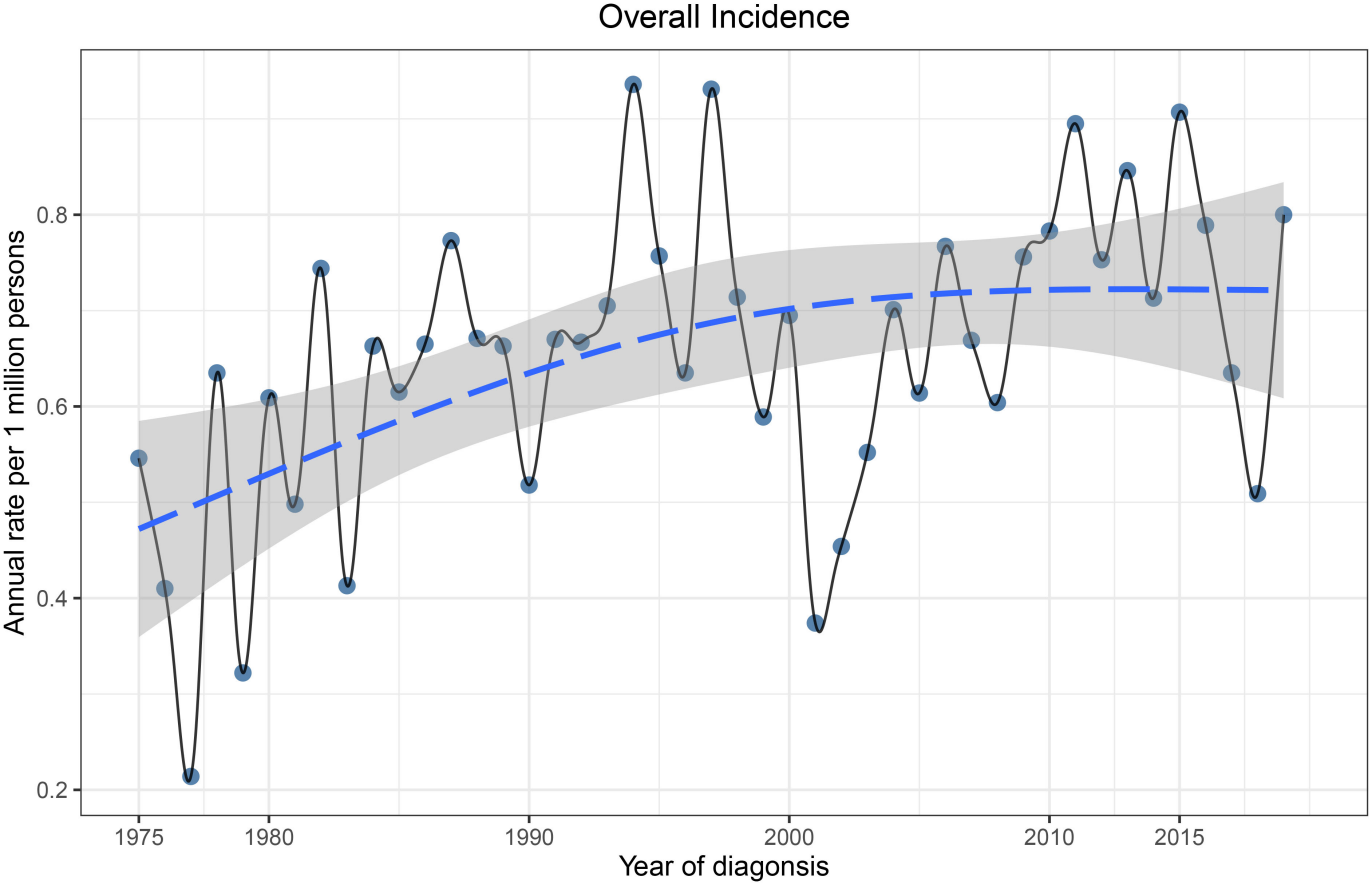
Incidence of primary orbital region ncSCC from 1975 to 2019 adjusted to the 2000 standard United States population. ncSCC, non-cutaneous squamous cell carcinoma.

The total incidence of ncSCC of the orbital region from 1975 to 2019 was 0.68 per 1,000,000 persons (APC, 0.69; 95% CI, 0.184–1.192; p < 0.05), with the incidence steadily increasing during this period (**Figure 3**). The incidence of ncSCC of the orbital region in men (1.16) was significantly higher than that in women (0.32). In addition, the incidence in people aged ≥60 years (2.86) was higher than in those aged <60 years (0.25), and the difference was statistically significant. Furthermore, the incidence of ncSCC of the orbital region in White people was 0.72 (APC, 0.85; 95% CI, 0.297–1.409; p < 0.05), which was significantly higher than the incidence in other races (Black, American Indian/AK Native, Asian/Pacific Islander, and race unknown). The most common primary site for ncSCC of the orbital region was the conjunctiva (0.5), followed by the orbit, lacrimal apparatus, and overlapping lesion of the eye and adnexa (**Table 1**).

**Table 1:** Incidence rate from 1975 to 2019.

	Incidence rate	APC	Incidence rate ratio (95% CI)	p
**Overall**	0.678	0.687* (0.184–1.192)		
Age				
<60	0.246	NA	Ref	
≥60	2.861	0.512 (−0.17 to 1.199)	11.459 (9.69–13.551)	<0.001
Sex				
Male	1.164	0.186 (−0.54 to 0.917)	Ref	
Female	0.319	NA	0.346 (0.291–0.413)	<0.001
Race				
White	0.719	0.851* (0.297 to 1.409)	Ref	
Others^a^	0.475	NA	0.496 (0.391–0.628)	<0.001
Primary site				
Conjunctiva	0.503	0.158 (−0.512 to 0.832)	Ref	
Lacrimal gland	0.066	NA	0.132 (0.101–0.171)	<0.001
Orbit	0.086	NA	0.167 (0.132–0.212)	<0.001
Overlapping lesion of eye and adnexa	0.023	NA	0.044 (0.028–0.068)	<0.001

APC, annual percentage change. NA, not applicable.

Others^a^: Black, American Indian/AK Native, Asian/Pacific Islander, and unknown.

### 3.2 Demographics of ncSCC of the orbital region

A total of 1,265 patients were included in this study. The mean age of the patients at the time of diagnosis was 65.3 ± 15.3 years (range, 6–101 years). Of 1,265 included patients, 823 (65.1%) were aged ≥60 years. The average age of patients with lacrimal apparatus SCC was much younger (59.9 years) than the overall average age of the patients. The proportion of men (73.5%) in the study population was significantly higher than that of women (26.5%). Regarding race, White people accounted for 87.4% of the study population, whereas other races accounted for only 12.6%. Regarding primary tumor sites, the conjunctiva (74.5%) was the most common site, followed by the orbit (12.1%), lacrimal apparatus (10.8%), and overlapping lesion of the eye and adnexa (2.7%). Regarding the SEER summary stage, most patients had localized cancer (81.5%), followed by regional (15.2%) and distant (3.3%) cancers. A total of 877 (69.3%) patients underwent surgery, and 14.8% and 8.5% received chemotherapy and radiotherapy, respectively. Patients with lacrimal apparatus SCC (66.2%) and orbital SCC (45.1%) were more likely to undergo radiotherapy than those with SCC at other sites. The detailed characteristics of the patients are presented in **Table 2**.

**Table 2:** Demographic and clinical characteristics of patients.

Variables	Total	Conjunctiva	Lacrimal apparatus	Orbit	Overlapping lesion of eye and adnexa	p
Number of patients (%)	1265	942 (74.5%)	136 (10.8%)	153 (12.1%)	34 (2.7%)	
**Age**						
Mean (SD)	65.3 (15.3)	64.5 (15.4)	59.9 (12.2)	73.2 (13.9)	72.9 (13.3)	
Median [min, max]	66 [6, 101]	66 [6, 98]	59.5 [35, 87]	77 [41, 101]	76 [44, 96]	
<60	442 (34.9%)	337 (35.8%)	68 (50%)	32 (20.9%)	5 (14.7%)	<0.001
≥60	823 (65.1%)	605 (64.2%)	68 (50%)	121 (79.1%)	29 (85.3%)	
**Year of diagnosis**						0.258
1975–1985	74 (5.8%)	58 (6.2%)	5 (3.7%)	8 (5.2%)	3 (8.8%)	
1986–1996	176 (13.9%)	132 (14%)	14 (10.3%)	21 (13.7%)	9 (26.5%)	
1997–2007	416 (32.9%)	307 (32.6%)	43 (31.6%)	54 (35.3%)	12 (35.3%)	
2008–2018	599 (47.4%)	445 (47.2%)	74 (54.4%)	70 (45.8%)	10 (29.4%)	
**Sex**						<0.001
Male	930 (73.5%)	718 (76.2%)	82 (60.3%)	101 (66%)	29 (85.3%)	
Female	335 (26.5%)	224 (23.8%)	54 (39.7%)	52 (34%)	5 (14.7%)	
**Race**						<0.001
White	1106 (87.4%)	832 (88.3%)	103 (75.7%)	143 (93.5%)	28 (82.4%)	
Others^a^	159 (12.6%)	110 (11.7%)	33 (24.3%)	10 (6.5%)	6 (17.6%)	
**Marital status**						0.118
Married	677 (53.5%)	515 (54.7%)	76 (55.9%)	68 (44.4%)	18 (52.9%)	
Other	588 (46.5%)	427 (45.3%)	60 (44.1%)	85 (55.6%)	16 (47.1%)	
**SEER summary stage**						<0.001
Localized	1031 (81.5%)	876 (93%)	57 (41.9%)	65 (42.5%)	33 (97.1%)	
Regional	192 (15.2%)	64 (6.8%)	59 (43.4%)	68 (44.4%)	1 (2.9%)	
Distant	42 (3.3%)	2 (0.2%)	20 (14.7%)	20 (13.1%)	0	
**Surgery**						0.244
Performed	877 (69.3%)	658 (69.9%)	100 (73.5%)	98 (64.1%)	21 (61.8%)	
No/unknown	388 (30.7%)	284 (30.1%)	36 (26.5%)	55 (35.9%)	13 (38.2%)	
**Radiotherapy**						<0.001
Performed	187 (14.8%)	27 (2.9%)	90 (66.2%)	69 (45.1%)	1 (2.9%)	
No/unknown	1078 (85.2%)	915 (97.1%)	46 (33.8%)	84 (54.9%)	33 (97.1%)	
**Chemotherapy**						0.004
Performed	107 (8.5%)	65 (6.9%)	16 (11.8%)	23 (15%)	3 (8.8%)	
No/unknown	1158 (91.5%)	877 (93.1%)	120 (88.2%)	130 (85%)	31 (91.2%)	

SEER, Surveillance, Epidemiology, and End Results.

Others^a^: Black, American Indian/AK Native, Asian/Pacific Islander, and unknown.

### 3.3 Survival analysis

**Figure 4:**
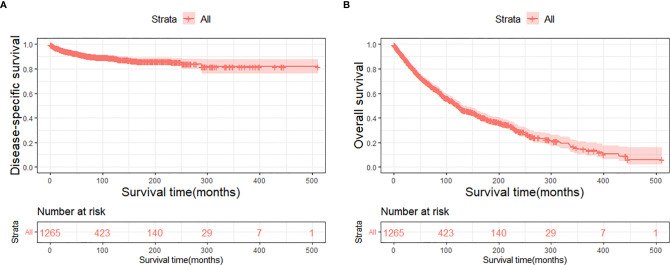
Disease-specific survival **(A)** and overall survival **(B)** of primary orbital region ncSCC for all patients. ncSCC, non-cutaneous squamous cell carcinoma.

**Figure 5:**
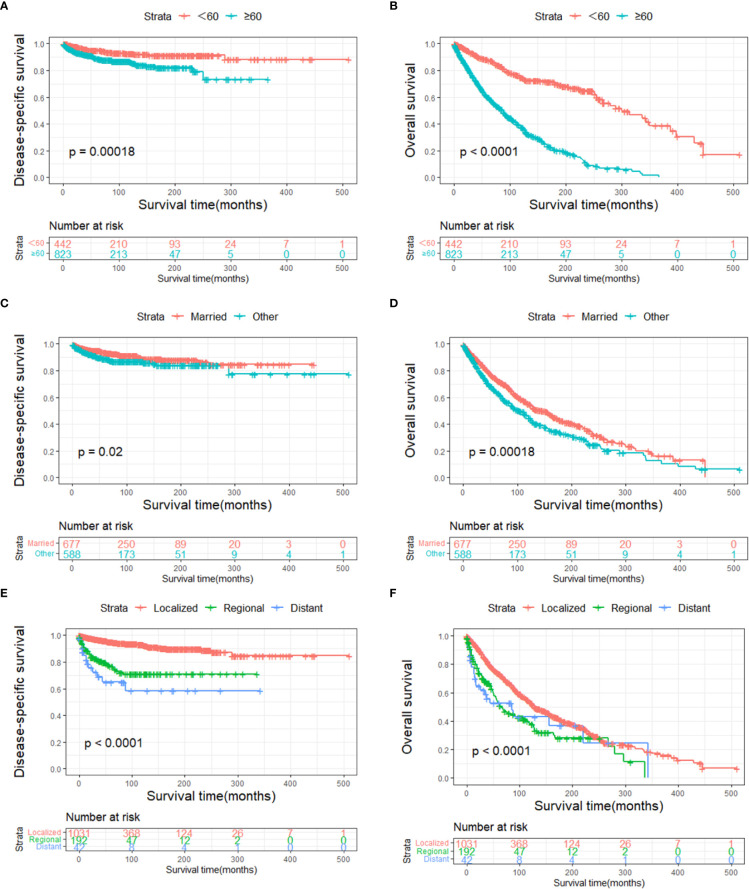
Disease-specific survival according to **(A)** age, **(C)** marital status, and **(E)** stage, and overall survival according to **(B)** age, **(D)** marital status, and **(F)** stage.

**Figure 6:**
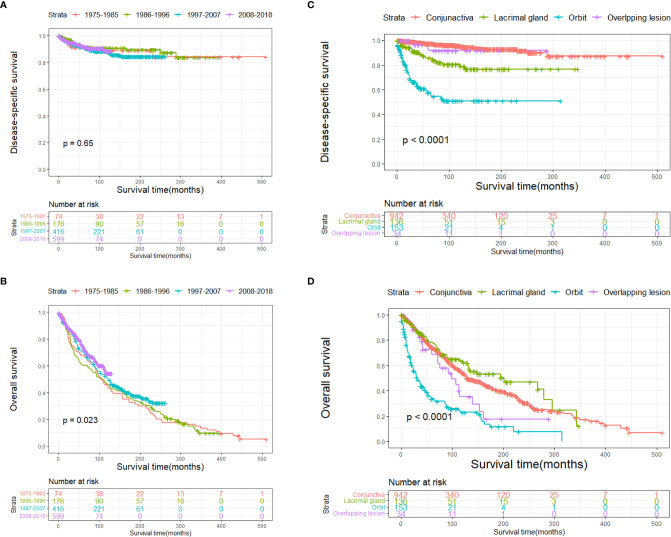
Disease-specific survival according to **(A)** year and **(C)** primary site, and overall survival according to **(B)** year and **(D)** primary site.

**Figure 7:**
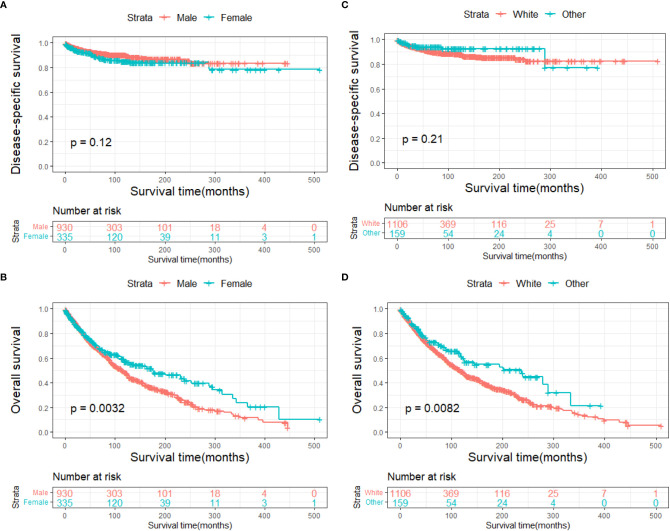
Disease-specific survival according to **(A)** sex and **(C)** race, and overall survival according to **(B)** sex and **(D)** race.

**Figure 8:**
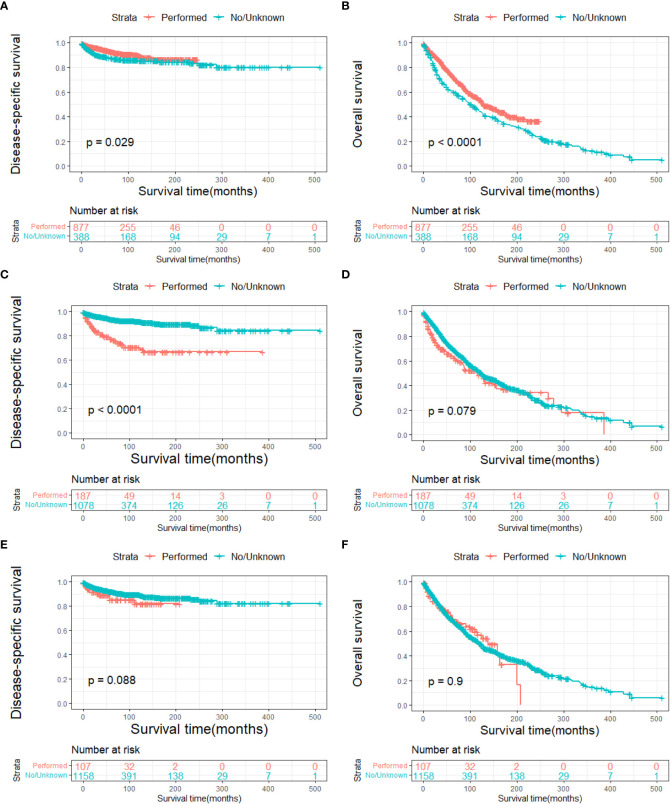
Disease-specific survival according to **(A)** surgery, **(C)** radiotherapy, and **(E)** chemotherapy, and overall survival according to **(B)** surgery, **(D)** radiotherapy, and **(F)** chemotherapy.

The DSS and OS rates of all the patients are shown in **Figures 4A, B**, respectively. A total of 610 patients died by the end of the observation period; of these, 114 died of SCC. The Kaplan–Meier survival analysis showed that the 1-, 5-, and 10-year DSS rates of ncSCC of the orbital region were 97.2%, 91.4%, and 88.8%, respectively, whereas its 1-, 5-, and 10-year OS rates were 93%, 70%, and 50.8%, respectively. The survival plots for age, year of diagnosis, sex, race, marital status, primary site, SEER summary stage, and treatment methods were constructed based on the results of the Kaplan–Meier analysis. Patients younger than 60 years, married patients, and those with the localized disease had better DSS and OS than those older than 60 years, unmarried patients, and those with other SEER summary stages (**Figures 5A–F**). Patients who were diagnosed later appeared to show prolonged survival (**Figures 6A, B**). Patients with orbital SCC had significantly reduced DSS and OS compared with those with SCC of the other primary sites (**Figures 6C, D**). Men and White people had a shorter OS than women and people from other races. However, there was no significant difference in DSS between these sex and race groups (**Figures 7A–D**). Regarding treatment modalities, patients who underwent surgery showed significantly improved DSS and OS (**Figures 8A, B**), whereas radiotherapy and chemotherapy were not associated with a better prognosis (**Figures 8C–F**).

A Cox proportional hazards model was used to identify the independent prognostic factors for DSS and OS in patients with ncSCC of the orbital region. The analysis revealed that age, primary site, SEER summary stage, and surgery independently predicted DSS (**Table 3**), whereas age, sex, marital status, primary site, SEER summary stage, and surgery independently predicted OS (**Table 4**).

**Table 3:** The results of the univariate and multivariate Cox regression analysis for DSS.

Variables	Univariate analysis		Multivariate analysis	
	HR (95% CI)	p-Value	HR (95% CI)	p-Value
**Age**		<0.001		0.001
<60	Ref		Ref	
≥60	2.264 (1.46–3.511)		2.103 (1.341–3.298)	
**Year of diagnosis**		0.647		
1975–1985				
1986–1996				
1997–2007				
2008–2018				
**Sex**		0.117		
Male				
Female				
**Race**		0.21		
White				
Others^a^				
**Marital status**		0.021		0.14
Married	Ref			
Other	1.546 (1.069–2.237)			
**Primary site**		<0.001		<0.001
Conjunctiva	Ref		Ref	
Lacrimal apparatus	4.273 (2.514–7.263)	<0.001	3.253 (1.793–5.902)	<0.001
Orbit	15.326 (9.995–23.498)	<0.001	10.434 (6.413–16.977)	<0.001
Overlapping lesion of eye and adnexa	1.549 (0.373–6.437)	0.547	1.335 (0.321–5.559)	0.691
**SEER summary stage**		<0.001		0.012
Localized	Ref		Ref	
Regional	4.667 (3.133–6.951)	<0.001	1.901 (1.202–3.006)	0.006
Distant	7.175 (3.936–13.077)	<0.001	2.044 (1.061–3.939)	0.033
**Surgery**		0.03		0.029
Performed	Ref		Ref	
No/unknown	1.518 (1.041–2.214)		1.528 (1.045–2.236)	
**Radiotherapy**		<0.001		0.932
Performed	Ref			
No/unknown	0.255 (0.175–0.374)			
**Chemotherapy**		0.088		
Performed				
No/unknown				

DSS, disease-specific survival; SEER, Surveillance, Epidemiology, and End Results.

Others^a^: Black, American Indian/AK Native, Asian/Pacific Islander, and unknown.

**Table 4:** The results of the univariate and multivariate Cox regression analysis for OS.

Variables	Univariate analysis		Multivariate analysis		
		HR (95% CI)	p-Value	HR (95% CI)	p-Value
**Age**		<0.001		<0.001
<60	Ref		Ref	
≥60	4.14 (3.346–5.123)		3.961 (3.194–4.913)	
**Year of diagnosis**		0.025		0.15
1975–1985	Ref			
1986–1996	1.015 (0.755–1.363)	0.923		
1997–2007	0.85 (0.641–1.127)	0.26		
2008–2018	0.709 (0.521–0.964)	0.028		
**Sex**		0.003		<0.001
Male	Ref		Ref	
Female	0.749 (0.618–0.909)		0.686 (0.561–0.839)	
**Race**		0.009		0.451
White	Ref			
Others^a^	0.694 (0.528–0.912)			
**Marital status**		<0.001		<0.001
Married	Ref		Ref	
Other	1.353 (1.154–1.586)		1.388 (1.179–1.634)	
**Primary site**		<0.001		<0.001
Conjunctiva	Ref		Ref	
Lacrimal apparatus	0.832 (0.621–1.116)	0.22	0.827 (0.598–1.145)	0.252
Orbit	3.189 (2.579–3.943)	<0.001	2.479 (1.944–3.162)	<0.001
Overlapping lesion of eye and adnexa	1.51 (0.974–2.342)	0.066	1.171 (0.755–1.818)	0.481
**SEER summary stage**		<0.001		0.001
Localized	Ref		Ref	
Regional	1.678 (1.359–2.072)	<0.001	1.623 (1.277–2.063)	<0.001
Distant	1.677 (1.103–2.548)	0.016	1.244 (0.787–1.968)	0.35
**Surgery**		<0.001		0.002
Performed	Ref		Ref	
No/unknown	1.396 (1.181–1.651)		1.304 (1.102–1.542)	
**Radiotherapy**		0.08		0.545
Performed				
No/unknown				
**Chemotherapy**		0.899		
Performed				
No/unknown				

OS, overall survival; SEER, Surveillance, Epidemiology, and End Results.

Others^a^: Black, American Indian/AK Native, Asian/Pacific Islander, and unknown.

## 4 Discussion

Owing to the rarity of ncSCC of the orbital region, very few studies on its incidence, clinicopathological features, and prognosis are available. Hence, we conducted a population-based cohort study to analyze the epidemiology and survival rates of ncSCC of the orbital region using the SEER database. In addition, we calculated the overall incidence and incidence trends of the disease and described its characteristics, treatment strategies, and its independent prognostic factors. To our knowledge, this is the first study to describe the demographic characteristics and prognosis of ncSCC of the orbital region in a large population.

The present study revealed that despite the rarity of ncSCC of the orbital region, its incidence has been increasing over the last 40 years, with an overall incidence of 0.68/1,000,000 persons. Previous reports have indicated that the incidence of conjunctival SCC has been increasing over years as well (14, 20, 21). The etiology of conjunctival SCC is associated with ultraviolet radiation, vitamin A deficiency, the human immunodeficiency virus, and the human papillomavirus, which also contributes to the etiology of the lacrimal drainage system SCC (22–24). It is speculated that the increasing incidence of conjunctival SCC may be attributed to the prolonged life expectancy of people with HIV infections owing to the advancement of diagnostic and therapeutic tools and methods (20, 25).

The average age of the patients included in this study was 65.3 years. This is consistent with the average age of patients with cutaneous SCC reported in a previous study (26). The results of the present study indicated that elderly patients were more likely to have ncSCC of the orbital region than younger patients and that advanced age was associated with worse DSS and OS. This may be because elderly persons generally have more comorbidities than younger people, which may affect their survival and treatment regimen (27). The incidence of ncSCC of the orbital region in men was significantly higher than that in women. This finding is attributable to the greater exposure to sunlight among men with conjunctival SCC (28, 29). The incidence in White people was higher than that in other races. Large genome-wide association studies conducted in non-Hispanic Whites revealed 10 susceptibility loci and partly explained inherited SCC susceptibility. However, their influence on ncSCC needs further study (30). In the present study, the conjunctiva was the most common primary site, followed by the orbit and lacrimal apparatus. In addition, patients with conjunctival SCC showed prolonged survival compared with those with SCC of the other primary sites. Orbital SCC was rare and had the worst prognosis, probably because of its atypical pathological process and relatively long course from symptom onset to diagnosis (10, 31). Furthermore, the average age of the patients with orbital SCC was older than that of the patients with SCC at other sites. This may have played a role in the poor prognosis of the patients with orbital SCC. Regarding the disease stage, we used the SEER summary stage instead of the TNM stage, which is more commonly used in clinical settings. This is because complete TNM staging data on ncSCC of the orbital region were unavailable in the SEER database. However, SEER summary staging includes a set of standardized and simplified coding rules for maintaining consistent definitions over decades (32). The multivariate Cox regression analyses conducted in the present study revealed that the SEER summary stage was an independent prognostic factor for both DSS and OS. This result is in line with those of previous studies that demonstrated that the advanced disease stage is associated with a high rate of recurrence and a worse prognosis (6, 33).

Surgical excision is the primary treatment for SCC of the conjunctiva, orbit, and lacrimal apparatus. In the present study, survival analysis revealed that surgery was an independent protective factor for DSS and OS. Conjunctival SCC usually involves the cornea. For relatively small tumors that involve less than one-third of the corneal limbus or 15 mm of the basal dimension, surgical excision with cryotherapy is an appropriate treatment option. To decrease the rate of microscopic seeding, several techniques are applied, including the “no touch technique” with a margin of 3–4 mm, independent use of surgical instruments inside and outside the cut margin, and a dry surgical field (34–36). For large or invasive tumors, extensive surgery, including radical surgery and enucleation, should be performed (34, 37, 38). Apart from surgery, several topical treatments, including mitomycin-C, interferon-α2b, and 5-fluorouracil, have been used in patients who show good compliance to avoid the risks of surgery and especially for tumors involving the cornea (39, 40). Surgery is also the first-line treatment for SCC of the lacrimal apparatus. Adjuvant radiotherapy is considered depending on the extent and size of the tumor, as well as the general health of the patient (41). However, the prognosis of patients who undergo globe-preserving excision and orbital exenteration remains controversial (11). Total exenteration disfigures the face and causes vision loss, which ultimately reduces the patient’s quality of life (42). Due to the lack of clear evidence that orbital exenteration improves the survival of patients with lacrimal apparatus carcinoma, eye-sparing surgical excision should be considered for localized and small tumors, whereas orbital exenteration may be considered for advanced cases (6, 43, 44).

Typically, there is no squamous epithelium in the orbit. One plausible theory for primary orbital SCC is that epithelial cells are occasionally trapped in the orbit during embryogenesis and gradually progress to malignancy (31). Two cases of orbital SCC that developed from transplantation and malignant transformation of the conjunctival epithelium caused by retinal surgery have been reported and may be a potential pathogenesis (9, 45). Due to the rarity of orbital SCC, there are no data on its survival rates or standard treatment. In the present study, patients with primary orbital SCC had the worst prognosis; however, surgery prolonged their survival.

The multivariate Cox regression analysis conducted in the present study showed that neither radiotherapy nor chemotherapy was a risk factor for orbital SCC. This may have been caused by the bias in the selection of treatments for various patients. In conjunctival SCC, brachytherapy is used as an adjuvant therapy for tumors with deep margins, and external beam radiotherapy is administered for the palliation of extensive carcinomas, including tumors that invade the eyeball (34, 38). Murthy et al. and El-Assal et al. reported complete resolution after radiotherapy with few side effects (46, 47). Several novel therapies have been used to treat conjunctival SCC. Among these, epidermal growth factor receptor inhibitors and checkpoint inhibitors have shown great promise (38). For SCC of the lacrimal apparatus, postoperative adjuvant radiotherapy is recommended for aggressive tumors. In addition, radiation allows for local control of the tumor and decreases the risk of recurrence; however, whether it prolongs the survival of patients requires further study (44). Owing to the relatively poor survival of patients with tumors of the lacrimal apparatus, chemotherapy is generally added to their treatment regimen. In addition, platinum compounds are usually used in adjuvant chemoradiation for head and neck SCC to increase the radiosensitivity of the treatment (11, 44, 48). Tse et al. reported that intra-arterial chemotherapy improves the prognosis of patients with carcinoma of the lacrimal apparatus; however, further study is required to validate its treatment effect (49).

This study has several limitations. First, all data used in this study were extracted from the SEER database. This may have led to inevitable biases, as in other retrospective studies. In addition, detailed information on some variables, such as radiotherapy and chemotherapy regimens, was missing. Second, owing to the rarity of ncSCC of the orbital region, we included the data of patients diagnosed between 1975 and 2018. This may have been responsible for the lack of complete data on the TNM stage, which is used more commonly in clinical settings. Thus, we used the SEER summary stage instead to maintain consistency in the extracted data. Third, in the SEER coding scheme, the primary site is used to describe a tumor developing in one site and not extending or metastasizing to other sites. However, we could not ascertain whether all the tumors did not extend from the surrounding tissue of the sinus or orbit. Fourth, the exact age of patients aged over 100 years old is not provided in the SEER; thus, we used 101 to denote patients older than 100 years. Nonetheless, the SEER program remains a valuable tool for studying rare tumors.

## 5 Conclusion

The present study demonstrates that the incidence of ncSCC of the orbital region, which is very rare, showed an increasing trend from 1975 to 2019. The results also showed that ncSCC of the orbital region usually affects White people, men, and people aged ≥60 years, and its most common site is the conjunctiva. Age, primary site, SEER summary stage, and surgery were independent prognostic factors for the survival of patients with ncSCC of the orbital region. The results of this study may provide new insights into improving the healthcare and management of patients with ncSCC of the orbital region.

## Data availability statement

The datasets presented in this study can be found in online repositories. The names of the repository/repositories and accession number(s) can be found below: Surveillance, Epidemiology, and End Results (SEER) Program (www.seer.cancer.gov) SEER*Stat Database: Incidence - SEER Research Data, 8 Registries, Nov 2021 Sub (1975-2019) - Linked To County Attributes - Time Dependent (1990-2019) Income/Rurality, 1969-2020 Counties, National Cancer Institute, DCCPS, Surveillance Research Program, released April 2022, based on the November 2021 submission.

## Ethics statement

The protocol was approved by the Ethics Committee of Changzheng Hospital. The patient in this research provided informed consent.

## Author contributions

R-lW designed and supervised the study. L-fH and PM wrote the manuscript. All authors contributed to the article and approved the submitted version.
